# Radiologisch-nuklearmedizinische Hybriddiagnostik mit SPECT/CT bei chronischen Fuß- und Sprunggelenkserkrankungen: Eine neue und wichtige Erweiterung des diagnostischen Spektrums

**DOI:** 10.1007/s00132-023-04377-3

**Published:** 2023-04-27

**Authors:** Hans Christian Rischke, Charlotte Hase, Thomas Schneider, Markus Walther

**Affiliations:** 1 SPECT/CT-Institut Freiburg, Schwabentorplatz 6, 79098 Freiburg, Deutschland; 2 Fuß- und Sprunggelenkchirurgie, Loretto-Krankenhaus Freiburg, Freiburg, Deutschland; 3 Sportmedizin, Fuß- und Sprunggelenkchirurgie, Gelenkklinik Gundelfingen, Gundelfingen, Deutschland; 4 Zentrum für Fuß- und Sprunggelenkchirurgie, Schön Klinik München-Harlaching, München, Deutschland

**Keywords:** Arthrose, Computertomographie, MRT, Fußknochen, Schmerzen, Szintigraphie, Arthrosis, Computed tomography, MRI, Foot bones, Pain, Scintigraphy

## Abstract

**Hintergrund und Ziel der Arbeit:**

Zahlreiche Erkrankungen des Fußes bzw. der Sprunggelenke können mithilfe der SPECT/CT (Single-Photon-Emmissions-Computertomographie/Computertomographie) im Hinblick auf ihre klinische Relevanz mit hoher Genauigkeit (bis > 90 %) diagnostiziert werden. Ziel dieser Übersichtsarbeit ist es, einen aktuellen Überblick über den Stellenwert der SPECT/CT bei ausgewählten Erkrankungen an Fuß- und Sprunggelenken zu geben, insbesondere in Abgrenzung zu MRT (Magnet-Resonanz-Tomographie), CT und Röntgen.

**Material und Methoden:**

Es erfolgte einer Literaturrecherche in der Datenbank Pubmed mit folgenden Begriffen: SPECT/CT, SPECT, skeletal or bone scintigraphy, CT, computed tomography, foot‑, ankle disease, ankle, tarsal, foot pain. Die Arbeiten wurden im Hinblick auf häufig auftretende Fragestellungen und Diagnosen selektiert. Ausgewählt wurden Arbeiten, die aufgrund der Anwendung der SPECT/CT eine genauere Diagnose verglichen zu alternativen Verfahren mit Änderung der Therapie beschreiben.

**Ergebnisse:**

In mehreren Studien wurde nachgewiesen, dass ein fokal erhöhter Knochenmetabolismus bei Arthrose und Osteochondrose signifikant mit der Schmerzentstehung korreliert. Die Präsenz von symptomatischen Ossikeln, wie zum Beispiel Os naviculare accessorium Typ II und Os trigonum, können mithilfe der SPECT/CT eindeutig nachgewiesen und mit keinem anderen bildgebenden Verfahren so treffsicher als Symptomquelle zugeordnet werden. Auch knöcherne Reaktionen im Bereich von Koalitionen, Arthrodesen, Osteosynthesen, okkulten Frakturen, Prothesen und beim diabetischen Fuß können mit keiner anderen bildgebenden Methode in vergleichbarer Genauigkeit nachgewiesen werden. Therapiekonzepte wurden bei unklaren Fällen, basierend auf Standardbildgebung inkl. MRT, durch die Zusatzinformation des SPECT/CT in bis zu zwei Drittel der Fälle geändert.

**Diskussion und Schlussfolgerung:**

Der Nutzen der SPECT/CT ist gegeben, wenn klinisch Unsicherheiten trotz Standardbildgebung bestehen.

## Abstract (en)

**Background and aim:**

In contrast to standard methods (sonography, X‑ray, CT (computed tomography), MRI (magnetic resonance imaging), numerous diseases of the foot or ankle can be diagnosed using SPECT/CT (single photon emission computed tomography/computed tomography) with regard to their clinical relevance with high accuracy (up to > 90%). The aim of this review is to provide an up-to-date overview of the importance of SPECT/CT in selected diseases of the foot and ankle.

**Material and methods:**

A literature search was carried out in the Pubmed database using the following terms: SPECT/CT, SPECT, skeletal scintigraphy, CT, computed tomography, foot and ankle disease, OSG, tarsal root, foot pain. The publications were selected with regard to questions and diagnoses that frequently occur in foot and ankle diagnostics. Furthermore, papers that describe a more precise diagnosis, a change in therapy management or a reduction in symptoms due to the use of SPECT/CT were selected.

**Results:**

Several studies have shown that a focally increased bone metabolism in osteoarthritis and osteochondral lesions correlates significantly with the development of pain. The presence of symptomatic ossicles such as the os naviculare accessorium type II and os trigonum can be clearly demonstrated with the help of SPECT/CT and cannot be assigned as the source of the symptoms as accurately with any other imaging method. Bony reactions in the area of coalitions, arthrodesis, osteosynthesis, occult fractures, prostheses and diabetic foot cannot be detected with comparable accuracy using any other imaging method, so that therapy concepts in unclear cases, based only on standard imaging are changed in up to 2/3 of cases by SPECT/CT information.

**Discussion and conclusion:**

SPECT/CT is useful when there are clinical uncertainties despite standard imaging.

## Einleitung und Hintergrund

Fuß- und Sprunggelenkschmerzen haben eine Prävalenz von 17–36 % in der Bevölkerung; Verletzungen in dieser Region gehören zu den häufigsten Krankheitsbildern in der Traumatologie [11, 23].

Im Fuß sind 28 Knochen, 33 Gelenke, ca. 107 Ligamente und 19 Muskeln und Sehnen auf engstem Raum vereint, welcher somit eine der komplexesten anatomischen muskuloskelettalen Regionen des Körpers darstellt. Es gibt zahlreiche Varianten wie akzessorische Knochen, Formabweichungen und Verschmelzungen von Fußwurzelknochen (Koalitionen) [5, 14]. Unterschiedliche Zug- und Druckkräfte wirken bei jeder Bewegung dreidimensional auf die verschiedenen Knochen und Gelenke. Störungen in diesem komplexen Gefüge wie Arthrosen, Arthritiden, kongenitale Fehlbildungen, Osteoporose oder Diabetes, können zu chronischen Beschwerden führen, die für den behandelnden Arzt eine Herausforderung darstellen. Klassische radiologische Verfahren wie Röntgen, Sonographie, CT und MRT haben Grenzen in der Darstellung der Ursache für chronische Schmerzen [5]. Ein besonderes Problem besteht, wenn gleichzeitig verschiedene Pathologien vorliegen. Gerade in diesen Situationen besteht der Bedarf nach weiteren präzisen bildgebenden Verfahren als Basis für eine sichere Diagnose und damit auch adäquate Therapie.

Eine Limitation der meisten bildgebenden Verfahren ist, dass lediglich die Morphologie und nicht die Funktion bzw. der Metabolismus des Gewebes dargestellt wird. Der Knochenmetabolismus repräsentiert die Antwort der Osteoblasten auf eine Belastung oder einen Reiz. Durch die Verwendung von knochenspezifischen szintigraphischen Tracern erlaubt die SPECT/CT (Single-Photon-Emmissions-Computertomographie/Computertomographie) den Grad der Osteoblastenaktivität mit hoher anatomisch-morphologischer Ortsauflösung darzustellen [12]. Die Tatsache, dass durch eine gleichzeitig akquirierte dünnschichtige Computertomographie die Skelettanatomie visualisiert wird, ermöglicht bei Kenntnis der spezifischen morphologischen radiologischen Zeichen von Skeletterkrankungen eine spezifische bzw. genaue Diagnose. Zum Beispiel können die bei Arthritiden typische gelenknahe Osteoporose, die Arrosion der „bare area“ an den Gelenkrändern, periartikuläre Verkalkungen oder feinfleckige Osteolysen und knöcherne Destruktionen bei Osteomyelitiden detektiert werden.

Diese der SPECT/CT einzigartige Eigenschaft hat dem Verfahren in den letzten 10 Jahren zu einem zunehmenden Erfolg in der nuklearmedizinisch-radiologischen Diagnostik verholfen. Eine wachsende Anzahl von Studien weist bei Fuß- und Sprunggelenkerkrankungen auf einen erheblichen Nutzen der SPECT/CT hin, insbesondere bei Patienten mit chronischen Fuß- und Sprunggelenksbeschwerden [4, 5, 9].

Als Radiotracer werden 99mTc-markierte Diphosphonate wie zum Beispiel 99mTc-DPD (99mTc‑3,3‑diphosphono‑1,2‑Propanodicarboxylicacid) verwendet. Die Strahlenexposition einer Fuß-SPECT/CT ist, insbesondere gemessen an der hohen Aussagekraft, als relativ niedrig einzustufen, und liegt in unserem Institut bei ca. 2,2 mSv (Millisievert), der Dosisanteil des CT beträgt 0,1 mSv. Zum Vergleich beträgt die effektive Dosis einer LWS-Röntgenaufnahme in 2 Ebenen ca. 0,6–1 mSv; die jährliche Strahlenexposition von Flugpersonal liegt bei ca. 2–5 mSv und die Maximaldosis bei beruflich strahlenexponierten Personen bei 20 mSV pro Jahr. Als einzige Kontraindikation gilt eine bestehende Schwangerschaft. Eine Fuß-SPECT/CT-Untersuchung erfolgt als 3‑Phasen-Skelettszintigraphie: in der ersten Phase wird die Durchblutung lokal und im Seitenvergleich gemessen, in der zweiten Phase können mit einer frühen SPECT pathologische Mehranreicherungen als Ausdruck entzündlicher Reizungen in den Weichteilen wie die Gelenkkapsel oder im Verlauf der Sehnen nachgewiesen werden. Nach 2 h werden der Knochenmetabolismus und die Morphologie mit der SPECT/CT dargestellt.

Ziel dieser Übersichtsarbeit ist es, einen aktuellen Überblick über den Stellenwert der SPECT/CT bei ausgewählten Erkrankungen an Fuß- und Sprunggelenken zu geben.

## Material und Methoden

Es erfolgte eine systematische Literaturrecherche in der Datenbank Pubmed mit folgenden Begriffen: SPECT/CT, SPECT, „skeletal or bone scintigraphy“, CT, „computed tomography“, „foot“, „ankle disease“, „ankle“, „tarsal“, „foot pain“. Die Arbeiten wurden im Hinblick auf in der Fuß- und Sprunggelenksdiagnostik häufig auftretende Fragestellungen und Diagnosen selektiert. Ausgewählt wurden Arbeiten, die aufgrund der Anwendung der SPECT/CT eine genauere Diagnose, eine Änderung des Therapiemanagements oder eine Beschwerdelinderung beschreiben. Die meisten publizierten Studien haben Fallzahlen im mittleren bis oberen zweistelligen bzw. im unteren dreistelligen Bereich, was auf die bisher begrenzte Verfügbarkeit dieser Diagnostik bzw. die Anwendung an spezialisierten Zentren zurückzuführen ist.

## SPECT/CT bei Arthrose

Die enge anatomische Beziehung der Mittel- und Rückfußgelenke bei gehäuft nur subtilen morphologischen Veränderungen, die jedoch Schmerzen verursachen können, kann mithilfe der SPECT/CT übersichtlich differenziert werden. Die gezielte Injektion von Lokalanästhetika in Fußwurzelgelenke mit dem höchsten Stoffwechsel im SPECT/CT ergab in mehreren Studien eine signifikante Beschwerdelinderung bei bis zu 90 % der behandelten Patienten und eine Änderung des Therapieplanes bei bis zu 78 % der Fälle [15, 22]. Der Vergleich der SPECT/CT-Befunde mit dem klinischen Verdacht, welche Gelenke schmerzverursachend sind, ergab 100 % Diskrepanz im Mittelfuß und 33 % Diskrepanz im Rückfuß [15]. Somit kann der Therapieerfolg einer Schmerzinfiltration mit dem SPECT/CT signifikant genauer vorhergesagt werden als mit einer klinischen Beurteilung. Es besteht eine hochsignifikante Korrelation (*p* < 0,001) der Intensität der Anreicherung des Radiopharmakons mit der Schmerz-VAS (Visuelle Analogskala) [30]. Bei der Planung von operativen Eingriffen am Sprunggelenk und Fuß liefert die SPECT/CT-Therapie entscheidende Informationen [6]. Im direkten Vergleich mit der MRT hat die SPECT/CT bei Arthrosen, insbesondere Fußwurzelarthrosen, eine deutlich höhere Spezifität als die MRT im Hinblick auf symptomatische Arthrosen, sodass in Kenntnis der SPECT/CT Therapiekonzepte häufig geändert werden [1, 4].

## SPECT/CT bei osteochondralen Läsionen

Osteochondrale Läsionen (OCL) sind Knorpelläsionen mit einem Defekt des darunterliegenden Knochens. Eine akkurate Diagnostik ist entscheidend für die Therapie. Neben der CT ist vor allem die MRT geeignet, um die lokalen Schäden darzustellen. Ein wesentlicher Schwachpunkt in der Darstellung osteochondraler Läsionen im MRT ist die unspezifische Darstellung von Knochenmarködemen. Die ossäre Pathologie wird im MRT daher häufig über-, seltener unterschätzt [10, 20]. Daher wird von einigen Autoren bei OCL grundsätzlich die Indikation zu einer SPECT/CT diskutiert [25]. Für die Genese des Schmerzes bei symptomatischen OCL ist nicht die Knorpelläsion, sondern die Reaktion im Knochen verantwortlich. Gelenkflüssigkeit gelangt durch den verletzten Knorpel unter Druck in den subchondralen Knochen, was eine lokale Osteolyse bzw. Zystenbildung erzeugt. Der lokale Überdruck und die lokale pH-Reduktion stimulieren die Nozizeptoren in den dicht innervierten subchondralen Knochenabschnitten [26].

**Figure 1:**
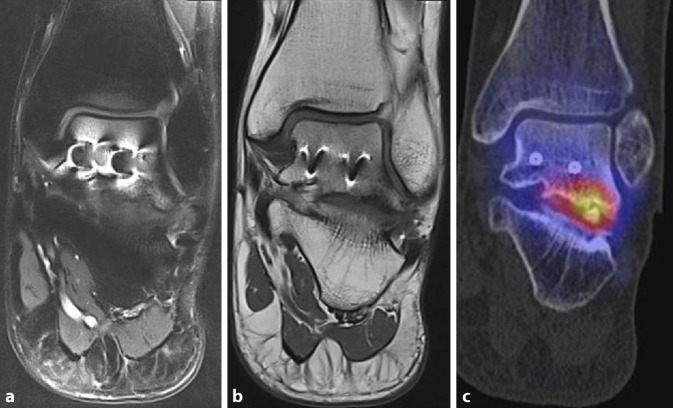

Studien zur Wertigkeit der SPECT/CT bei OCL ergaben eine sehr hohe Korrelation zwischen Knochenmetabolismus und Schmerzempfinden von bis zu 100 % [27]. Da insbesondere Patienten mit einem deutlichen Hypermetabolismus an der OCL von einer Operation profitieren, kommt dieser Information eine erhebliche therapeutische Bedeutung zu [20]. Ein Beispiel für eine symptomatische OCL zeigt Abb. 1: in der zum Vergleich mitabgebildeten MRT war die posttraumatische OCL nicht differenzierbar, was in diesem Fall auch durch Metallartefakte bedingt ist. Unklare Fälle mit Metallimplantaten sind für die SPECT/CT prädestiniert. Metallimplantate beeinträchtigen die Aussagekraft der SPECT/CT in aller Regel nicht.

## SPECT/CT bei akzessorischen Knochen und Normvarianten

Im Fußskelett finden sich zahlreiche Normvarianten, zu denen auch Varianten der Sesambeine, das Auftreten von Ossikeln und akzessorische Knochen gehören. Diese sind häufig asymptomatisch, können jedoch infolge von Überlastungen durch degenerative Veränderungen, mechanische Konflikte oder Traumata symptomatisch werden. Immer wieder sind akzessorische Knochen von Frakturen abzugrenzen. Für diese Fragestellungen ist die SPECT/CT eine optimale diagnostische Methode, was in verschiedenen Studien bestätigt wurde. Häufige akzessorische Knochen sind das Os naviculare accessorium (= Os tibiale externum, ca. 8 % Prävalenz) und das Os trigonum (ca. 10 % Prävalenz) [7, 14].

Bei einem symptomatischen Os naviculare accessorium Typ II erfolgt primär eine konservative Behandlung [2]. Eine operative Sanierung mit Entfernung des akzessorischen Knochens kommt bei persistierenden Beschwerden in Betracht. Eine Studie an 105 Patienten mit einer negativen Kontrollgruppe von 31 Patienten untersuchte, inwieweit der SPECT/CT-Metabolismus mit klinischen Beschwerden korreliert. Die Ergebnisse zeigten, dass Typ II mit einem hohen Tracer-Uptake an der Synchondrose häufiger reseziert werden mussten, um die Beschwerden zu lindern, sodass ein intensiv erhöhter Metabolismus bei Typ II ausschlaggebend für eine operative Sanierung sein kann [2]. Diese Ergebnisse wurden an einer retrospektiven multizentrischen Studie an 246 Patienten bestätigt [13].

**Figure 2:**
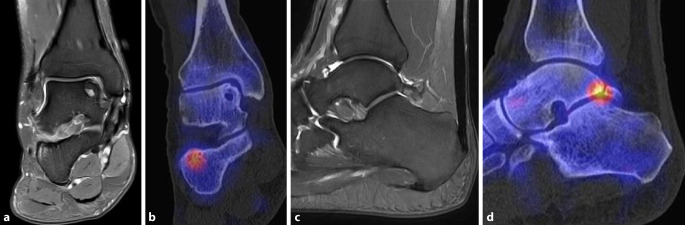

**Figure 3:**
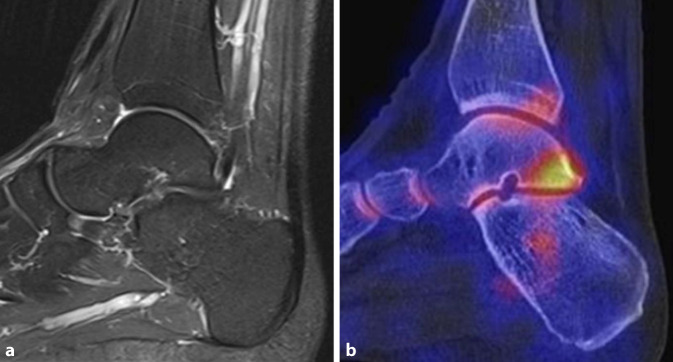

Ein weiteres Beispiel für den Einsatz der SPECT/CT ist das posteriore Impingement des Sprunggelenks. Das Impingement kann auch durch einen akzessorischen Knochen dorsal des Talus (Os trigonum) verursacht werden. Scherkräfte im Bereich der knorpeligen Verbindung zwischen Talus und Os trigonum lösen dabei eine hypermetabole Stressreaktion aus. Der Befund eines szintigraphisch aktivierten Os trigonum kann somit eine gezielte Intervention rechtfertigen [3]. Ein Beispiel für ein symptomatisches Os trigonum zeigt Abb. 2. Hier liegt gleichzeitig eine prominente osteochondrale Läsion der medialen Talusschulter vor, welche jedoch im SPECT/CT keinerlei Traceranreicherung zeigt und somit als „stumm“ und nicht symptomatisch einzustufen ist. Ein Beispiel für ein posteriores Impingement ohne Os trigonum zeigt Abb. 3, gegenüber dem MRT ist wie in Abb. 2 ein deutlicher fokaler Hypermetabolismus im SPECT/CT nachweisbar, der die Schmerzen erklärt.

## SPECT/CT bei Koalitionen und Arthrodesen

Koalitionen sind nichtgelenkige (knorpelige bis knöcherne) Verbindungen zwischen Fußwurzelknochen. Am häufigsten sind die Coalitio calcaneonaviculare und die Coalitio talocalcaneare mit einer Prävalenz von ca. 1 % [14].

**Figure 4:**
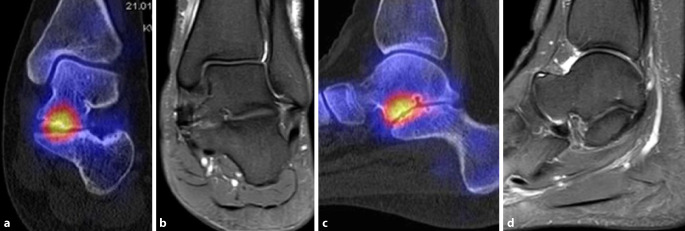

Koalitionen können auf Röntgenbildern wegen überlappender Strukturen teilweise nicht sicher diagnostiziert werden, sodass Schnittbildverfahren wie MRT und/oder CT erforderlich sind. Der Vorteil der SPECT/CT liegt darin, dass neben der Darstellung der Anatomie auch die Stressreaktion sichtbar gemacht werden kann. Dabei hat die SPECT/CT eine deutlich höhere Treffsicherheit als die MRT zur exakten Lokalisation der Stressreaktion [28]. Abb. 4 zeigt eine typische Coalitio talocalcaneare: der Hypermetabolismus im Bereich der Coalitio deutet darauf hin, dass vermehrte Scherkräfte auftreten, welche die Schmerzen des Patienten erklären können; das MRT dagegen ist hier unauffällig.

Arthrodesen gehören am Fuß zu den etablierten Therapieverfahren bei schmerzhaften Arthrosen. Eine zumindest teilweise knöcherne Durchbauung von Arthrodesen sollte in der Regel innerhalb von 6 Monaten postoperativ nachweisbar sein. Eine unvollständige oder fehlende Fusion nach Arthrodese kann über eine permanente Stressreaktion ähnlich wie bei Koalitiones persistierende Schmerzen verursachen, was sich mit der SPECT/CT nachweisen lässt. Studien zur Dauer einer erhöhten Osteoblastenaktivität nach Arthrodesen sind nicht publiziert. Unseren Erfahrungen nach ist 6–9 Monate nach Arthrodesenoperation häufig noch ein deutlich erhöhter Stoffwechsel bei nachweisbarer Durchbauung der Arthrodese zu sehen, weil die Osteoblasten noch aktiv sind; nach 1–1,5 Jahren sollte die Intensität der Anreicherung deutlich abgeklungen bzw. nur noch gering sein. Der Vorteil der SPECT/CT gegenüber der alleinigen CT bei persistierenden Beschwerden nach Arthrodesen ist, dass sowohl die CT-Morphologie als auch die Osteoblastenaktivität um die Arthrodese und in den Nachbargelenken untersucht wird. Somit können symptomatische Anschlussarthrosen nachgewiesen oder ausgeschlossen werden.

## SPECT/CT zur Beurteilung von Endoprothesen

**Figure 5:**
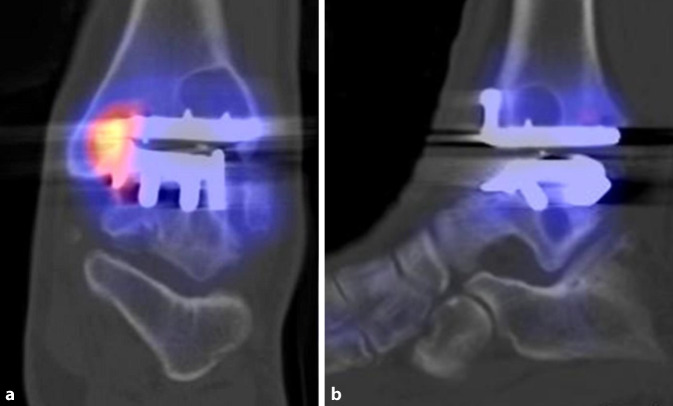

Weltweit nimmt die Anzahl der implantierten Sprunggelenksprothesen durch Weiterentwicklungen von Implantaten und chirurgischen Konzepten zu. Eine Herausforderung sind Patienten mit persistierenden Beschwerden nach Prothesenimplantation ohne klare Ursache [8]. Häufig ist es schwierig die Schmerzquelle mit konventionellen bildgebenden Verfahren (Röntgen, CT, MRT [auch mit Metallartefaktreduktion]) eindeutig zu identifizieren. Der diagnostische Wert der SPECT/CT ist bei liegender Sprunggelenkendoprothese analog der Beurteilung von anderen Prothesen (z. B. Knie und Hüfte) gegenüber den anderen bildgebenden Verfahren signifikant erhöht. Studien zeigten bis zu 89 % Korrelation des SPECT/CT mit intraoperativen Befunden bzw. der definitiven Diagnose [8] und eine Genauigkeit von 96 % (Sensitivität 100 %, Spezifität 80 %). Die Arbeitsgruppen von Mertens und Mason berichteten, dass bei schmerzhafter Sprunggelenkprothese in 86 % der Fälle der SPECT/CT-Befund die Grundlage für eine spezifische Therapie lieferte, mit einer Erfolgsrate von 83 % [19, 21]. Abb. 5 zeigt die Totalendoprothese eines Patienten mit medialem Impingement bei rezidivierender Varusfehlstellung nach Implantation einer Totalendoprothese des Oberen Sprunggelenks vor 4 Jahren.

## SPECT/CT beim diabetischen Fuß

Auch bei der Abgrenzung von Osteomyelitiden beim diabetischen Fußsyndrom stellt die SPECT/CT ein wertvolles Hilfsmittel dar, vor allem da die Diagnose Osteomyelitis im MRT zu häufig gestellt wird [16]. Durch die Kombination einer dünnschicht- bzw. hochauflösenden CT (< 1 mm Schichtdicke) mit einer SPECT/CT kann neben der entzündungsbedingten Knochenstoffwechselsteigerung die Morphologie der Entzündung detailliert dargestellt werden. Schätzungsweise 15–20 % der Diabetespatienten entwickeln im Verlauf ihrer Erkrankung ein Ulkus am Fuß [17, 24]. Diese Ulzera sind die primären Orte für die Entstehung von Infektionen und Osteomyelitiden. Bei optimaler Untersuchungstechnik liegt die Treffsicherheit zur Diagnostik von diabetischen Osteomyelitiden bei ca. 90 %, wobei insbesondere die SPECT/CT hilfreich ist, um die lokale Ausdehnung der Entzündung zu bestimmen bzw. um zwischen knöchernem und Weichteilbefall zu diskriminieren [17].

## SPECT/CT im direkten Vergleich mit der MRT

Studien, welche die diagnostische Aussagekraft der Skelettszintigraphie bzw. SPECT/CT direkt mit der MRT verglichen haben, weisen auf die höhere Spezifität der SPECT/CT im Hinblick auf die Schmerzursache bei verschiedenen Pathologien hin. Die Abb. 1, 2, 3, 4, 5 und 6 geben diese Beobachtungen beispielhaft wieder.

**Figure 6:**
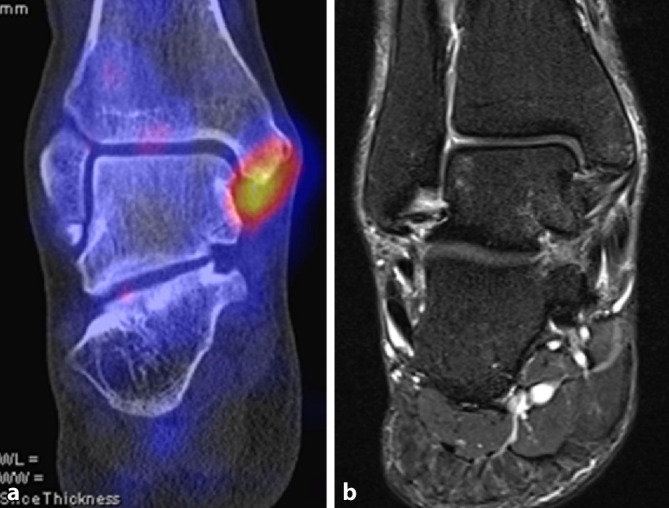

Ha et al. verglichen erstmals SPECT/CT systematisch mit MRT. Bei 50 Patienten mit 147 Läsionen an Knochen, Gelenken, Ligamenten und Sehnen wurden die Aussagen beider Verfahren analysiert. Bei Knochenläsionen und Ligament‑/Sehnenpathologien war die Spezifität des SPECT/CT mit 57 % vs. 10 % bzw. 73 % vs. 8 %, signifikant höher als die der MRT [9]. Eine andere Studie wies ebenfalls darauf hin, dass symptomatische Bandläsionen mit der SPECT/CT spezifisch nachgewiesen werden können [18]. Abb. 6 zeigt eine nur im SPECT/CT nachweisbare schmerzhafte Stressreaktion im Innenband (Deltaband) des oberen Sprunggelenkes.

Eine Erklärung für diese Beobachtungen liefert die Pathophysiologie der Tendinopathie. In Abhängigkeit vom Mikromilieus kommt es zu einer chondrogenen/osteogene Fehldifferenzierung der Sehnenstammzellen bzw. gewebsständigen Progenitorzellen. Dieser aktive Prozess kann mit szintigraphischen Tracern sichtbar gemacht werden [29].

In einer weiteren Studie wurde die diagnostische Wertigkeit von MRT und SPECT/CT bei Knöchel- und Fußschmerzen unklarer Ursache verglichen. Das Spektrum umfasste Weichteilläsionen (Bänder/Sehnen), Gelenkpathologien (Arthritis, Arthrose) und Knochenveränderungen (Frakturen/Osteomyelitis/osteochondrale Läsionen). Die doppelt so hohe Spezifität (60 % vs. 31 %) der SPECT/CT gegenüber der MRT führte in 26 % der Fälle zu einer Änderung der Therapie [1].

In einer weiteren Studie analysierten erfahrene Fuß- und Sprunggelenkchirurgen unabhängig klinische Daten und Röntgenaufnahmen zusammen mit MRT und/oder SPECT/CT. Eine Therapieempfehlung basierend auf einer SPECT/CT-Untersuchung wurde durch die weiteren Informationen einer MRT in 17 % geändert. Dagegen wurden Therapieempfehlungen basierend auf den Informationen einer MRT-Untersuchung nach Einbeziehung der Befunde des SPECT/CT in 60 % geändert. Die Autoren schlussfolgerten, dass die SPECT/CT einen hoch relevanten Einfluss auf die endgültige Therapiestrategie hat [4].

2020 publizierten Yeats et al. [28] ihre Ergebnisse bei Kindern und Jugendlichen: Das Durchschnittsalter der Patienten betrug 13,4 Jahre (Range 6–16,5 Jahre). Es handelte sich um 33 Patienten mit komplexen Fuß- und Knöchelschmerzen, die zwischen 2009 und 2019 zur SPECT/CT (insgesamt 36 Scans) überwiesen wurden. In 28 der 36 Fälle (77 %) lieferte die SPECT/CT therapieentscheidende Befunde, wobei 5 Operationen vermieden wurden. Die Autoren kamen zu dem Schluss, dass die SPECT/CT bei Kindern und Jugendlichen mit unklaren Fuß- und Sprunggelenksbeschwerden in vielen Fällen eine exakte Diagnose und damit auch eine suffiziente Therapie erlaubt. Dies war besonders eindrücklich bei Patienten mit mehrfach voroperiertem Fuß, akzessorischen Knochen und tarsalen Koalitiones. Insbesondere kann die SPECT/CT zwischen alten, therapierten (metabolisch inaktiven) Veränderungen und neu aufgetretenen (metabolisch aktiven) Pathologien differenzieren und hilft die Beschwerden einer spezifischen Veränderung zuzuordnen. Dabei stellen auch Metallimplantate kein Problem dar.

## Fazit für die Praxis

- Zahlreiche Erkrankungen des Fußes bzw. der Sprunggelenke können mit hoher Genauigkeit mithilfe der SPECT/CT (Single-Photon-Emmissions-Computertomographie/Computertomographie) diagnostiziert werden.
- Der Vorteil des Verfahrens ist besonders deutlich bei Fußwurzelarthrosen, osteochondralen Läsionen, Impingement des Sprunggelenks, Koalitiones, Stressfrakturen, Sesambeinpathologien, akzessorischen Knochen, Restbeschwerden nach Arthrodesen oder Sprunggelenkprothesen, Arthritiden, Osteomyelitis, diabetischem Fußsyndrom, Osteonekrosen sowie Sehnen- und Bandpathologien.
- Die SPECT/CT kann in unklaren und komplexen Fällen sowie beim Vorliegen von mehreren konkurrierenden Pathologien häufig entscheidende Informationen liefern, welche die weitere Therapie signifikant beeinflussen. Gerade in diesen Fällen ist der Einsatz der SPECT/CT aus dem Alltag des Fuß- und Sprunggelenkspezialisten nicht mehr wegzudenken.
